# Climate for Consumptives

**Published:** 1883-06

**Authors:** 


### HALL'S JOURNAL OF HEALTH

AND

MISCELLANY.

MONTHLY.

E. H. GIBBS, A. M., M. D., EDITOR.

FOUNDED IN 1854.

### CLIMATE FOR CONSUMPTIVES.

IF we take the opinions of medical men, and what is better still the experiences of consumptives themselves as to the value of any particular climate in this disease, we are forced to the conclusion that the best *climate* for a consumptive is that where he can enjoy the comforts of a good home, with opportunity for ample and proper exercise in the open air. A consumptive is not a fit subject for exile from home and friends; his spirits are depressed and he goes away feeling that he may not return, that the change is a forlorn hope, and the people he meets at the so-called "Health Resorts" have experiences to relate that are not calculated to inspire the new-comer with hope.

We believe that the larger proportion of consumptives who go from the North to Southern climes to spend the winter hasten the disease. No climate that depresses the most vigorous constitutions is fit for those who require the dry, tonic atmosphere of hills and mountains. There are a few rules that should be always observed by those who have any tendency to lung disease.

1st. Do not neglect the first symptoms of lung trouble. There is no disease that is more manageable if taken in time. Rest, warmth and almost entire abstinence from solid foods for 24 to 48 hours are generally sufficient to restore the usual health.

2d. Avoid medicines unless prescribed by a physician. The unskillful tampering with drugs is always fraught with danger.

3d. Should the cough and fever continue more than two or three days, call a physician. Neglect of these precautions has cost a world of suffering and millions of valuable lives.

The saddest of a physician's experiences is to be called to cases that have been rendered hopeless through neglect. Most of these cases are those that relate to lung diseases.

